# First-Time Migration in Juvenile Common Cuckoos Documented by Satellite Tracking

**DOI:** 10.1371/journal.pone.0168940

**Published:** 2016-12-22

**Authors:** Marta Lomas Vega, Mikkel Willemoes, Robert L. Thomson, Jere Tolvanen, Jarkko Rutila, Peter Samaš, Roine Strandberg, Tomáš Grim, Frode Fossøy, Bård Gunnar Stokke, Kasper Thorup

**Affiliations:** 1 Center for Macroecology, Evolution and Climate, Natural History Museum of Denmark, University of Copenhagen, Copenhagen, Denmark; 2 Department of Biology, University of Turku, Turku, Finland; 3 FitzPatrick Institute of African Ornithology, University of Cape Town, Cape Town, South Africa; 4 Department of Ecology, University of Oulu, Oulu, Finland; 5 Department of Environmental and Biological Sciences, University of Eastern Finland, Joensuu, Finland; 6 Department of Zoology and Laboratory of Ornithology, Palacky University, Olomouc, Czech Republic; 7 Department of Biology, Lund University, Lund, Sweden; 8 Department of Biology, Norwegian University of Science and Technology, Trondheim, Norway; 9 Norwegian Institute for Nature Research (NINA), Sluppen, Trondheim, Norway; Estacion Experimental de Zonas Aridas, SPAIN

## Abstract

Being an obligate parasite, juvenile common cuckoos *Cuculus canorus* are thought to reach their African wintering grounds from Palearctic breeding grounds without guidance from experienced conspecifics but this has not been documented. We used satellite tracking to study naïve migrating common cuckoos. Juvenile cuckoos left breeding sites in Finland moving slowly and less consistently directed than adult cuckoos. Migration of the juveniles (N = 5) was initiated later than adults (N = 20), was directed toward the southwest–significantly different from the initial southeast direction of adults–and included strikingly long Baltic Sea crossings (N = 3). After initial migration of juvenile cuckoos toward Poland, the migration direction changed and proceeded due south, directly toward the winter grounds, as revealed by a single tag transmitting until arrival in Northwest Angola where northern adult cuckoos regularly winter. Compared to adults, the juvenile travelled straighter and faster, potentially correcting for wind drift along the route. That both migration route and timing differed from adults indicates that juvenile cuckoos are able to reach proper wintering grounds independently, guided only by their innate migration programme.

## Introduction

Evidence about actual migration in solitary, nocturnal migrant birds is scarce and even mapping the spatiotemporal migration routes of individuals has only been accomplished for a few species [1]. Juvenile long-distance migrants of most nocturnal land-birds presumably migrate solitarily and without guidance from adults [2–4]. Documenting whether juvenile migrants travel independently of adults and whether they follow the same routes as adults has not been possible for smaller species because the necessary technology was unavailable [5]. Similarly, lack of suitable technology has hindered the study of how the inherited spatiotemporal guiding programme unfolds in free-flying individuals, for instance in route learning [6] and dealing with wind drift [7].

Common cuckoos *Cuculus canorus* are long-distance migrants wintering in Tropical Africa [8]. Because the cuckoo is an obligate brood parasite, there is presumably no contact between young and their biological parents after egg laying. It is thus assumed that soon after fledging, young cuckoos travel on their own [9], but to date this conjecture has not been supported with data. Therefore, young are thought to travel on their first migration without learning from experienced birds [10]. Of more than 19,000 common cuckoos ringed in Europe, only two birds ringed as young have been recorded from south of the Sahara (in Cameroon and Togo; [11]; S1 Table). Moreover, such single-point data provide no information on the route travelled.

Here, we map for the first time the movements and migration of juvenile cuckoos after fledging from nests in Finland using satellite-based radio telemetry. We describe the spatiotemporal progress of the first migration of the juveniles and compare this with the migration of experienced conspecifics. Our specific aims are to determine whether juvenile and adult cuckoos follow the same route and whether juveniles are potentially guided by experienced conspecifics on migration. We focus our analyses on spatiotemporal overlap in migration routes with adult cuckoos from nearby breeding grounds in North Scandinavia (northern adults; [12]). In addition, we investigate the spatiotemporal overlap with published tracks of adult cuckoos from South Scandinavia (southern adults; [13]), because our data showed that the initial migration of the juvenile cuckoos deviated from the northern adults, and that juveniles travelled toward stopover sites used by southern adults. To investigate spatiotemporal overlap between juveniles and adults, we specifically compare (i) initial migration direction, (ii) sea-crossing, (iii) geometry of tracks, including longitudes (northern adults only) and straightness (all adults), and (iv) timing of autumn migration periods.

## Methods

### Ethics statement

Catching and satellite tagging of cuckoos was permitted by Regional Environmental Centres in Ruokolahti (KASELY/353/07.01/2010) and Oulu (POPELY/166/07.01/2014) and by the Finnish Museum of Natural History (ringing permits 2784, 2836, 2973). Catching was carried out on private lands with permission from landowners. The study did not involve any endangered or protected species. Adults were caught with mist nets and young were taken in nest boxes. Blood sampling for sexing the cuckoos was permitted by the Finnish National Animal Experiment Board (permits ESAVI/2846/04.10.03/2012 and ESAVI/3221/04.10.07/2013). All sampling procedures and experimental manipulations were reviewed as part of obtaining the above-mentioned permits.

### Tracking data

#### Juvenile cuckoos

We fitted satellite transmitters (PTT-100 5 gram Solar PTT, Microwave Telemetry Inc.) on 13 fledgling common cuckoos during 2013–2014 at two Finnish breeding sites (S2 and S3 Tables)–nine young near Oulu in 2014 (65°N 25°50'E; [14]) and four young near Ruokolahti in 2013 (61°22'N 28°31'E; [15]). The young cuckoos at both sites were raised in nest boxes allowing close monitoring of their growth rates. The host species were common redstart *Phoenicurus phoenicurus* and in one case great tit *Parus major*. The young cuckoos were tagged just before fledging and the harness size was the same as the one used in adults to account for further body growth [13]).

**Fig 1:**
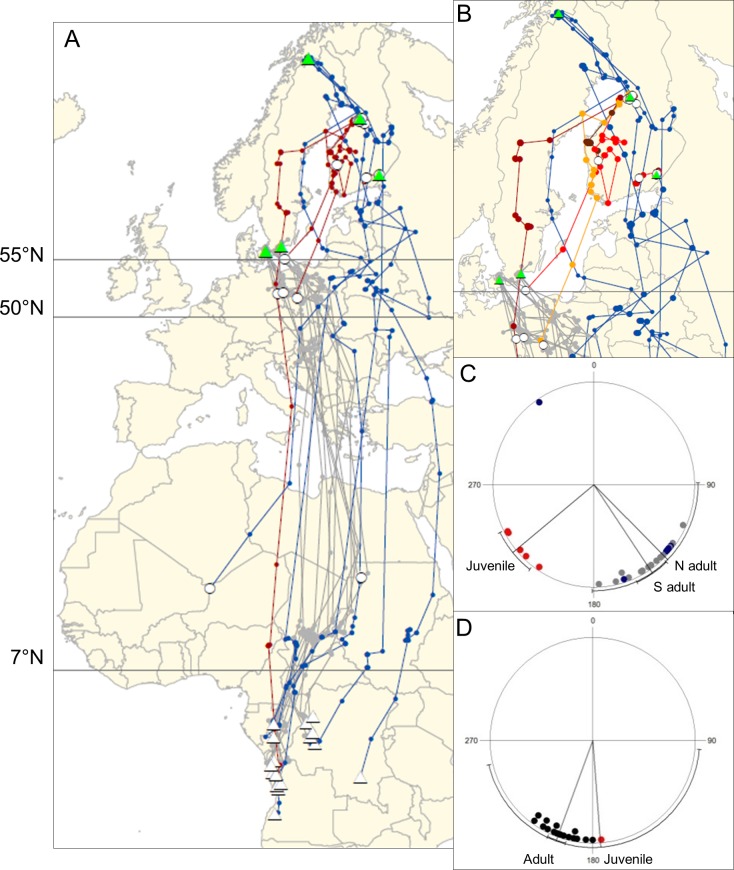
Autumn migration of common cuckoos. Tracks of juveniles from Finland (red), northern adults (N adults; North Sweden and Finland; blue) and southern adults (S adults; Denmark and South Sweden; grey) with tagging and wintering sites (green and white triangles, respectively), and end of transmission (white circles) indicated. (A) Migration from breeding to wintering grounds and (B) details of initial movements with different red tinge for each juvenile cuckoo. Best quality locations for each duty cycle (dots) connected with lines. Horizontal lines at 55°N and 50°N indicate the band used to define entering and leaving, respectively, the NC Europe stopover. The line at 7°N defines leaving the Sahel. (C) Initial travel directions from breeding sites after moving 100 km and (D) travel directions (N and S adults; black) after leaving the Sahel. Each dot indicates the direction of one individual. Lines are group mean directions with 95% confidence interval. Data on North Swedish adults from [12] and Danish and South Swedish adults from [13].

Of the 13 tagged nestlings, we consider 12 to have fledged successfully; for one, data was only received on the day of starting the transmitter indicating that fledging was not completed. Of the 12 successfully fledged juveniles, six transmitted positions for more than a month; four after 46 days and one the entire autumn migration, ceasing transmission after 12 days being stationary in the wintering grounds (Fig 1, S2 Table). Satellite data rarely allows distinguishing death from tag failure [16]. For one tag, transmissions indicated death and this tag was later recovered on the dead bird. No other tags allowed distinguishing the cause of the stopped transmission, but death is likely to be the most common cause. For the successfully fledged juveniles (N = 12), five (42%) transmitted until assumed independent (i.e. >20 km from nest). Of those reaching independence (N = 5), one (20%) transmitted until completion of autumn migration.

Survival of young cuckoos varies depending on the host [17, 18] and survival of cuckoos fledged from redstart nests has not been estimated before. Assuming that all lost tags stopped transmitting because of death, our survival rates from fledging to independence were lower than those reported by Wyllie [19], 42% versus 62%, but not significantly so (S4 Table). Survival of juveniles during the first migration (20%) was lower than those reported for adult satellite-tagged birds (down to 53% for UK birds travelling via the western route [16]), though not significantly lower (S4 Table) presumably due to the low sample sizes. Survival during the first migration is expected to be lower than during later migrations; in satellite-tracked Egyptian vultures *Neophron percnopterus*, survival during the first autumn migration was 47% compared to 95% in adults [20]. In cuckoos, Wyllie [19] reported 4 out of 60 (7%) tagged fledglings returning to their breeding site in the following year but the degree of natal dispersal in cuckoos is not known.

#### Adult cuckoo data

In addition to the data on juvenile cuckoos, we included tracks of adults from Finland and Scandinavia. Tracks of two adult cuckoos (two autumn migrations) from 2014, one caught near Oulu and one near Ruokolahti were included, as were published tracks of 11 adult birds (18 autumn migrations) from Scandinavia (Denmark: 10 tracks from four adults [13]; South Sweden: four tracks from four adults [13]; North Sweden: four tracks from three adults [12]). For comparison with juvenile birds, we lumped the adult tracks into two different groups–northern adults (North Swedish and Finnish) and southern adults (Danish and South Swedish). Transmission stopped in three birds before arrival south of the Sahara Desert (Fig 1, S2 Table).

### Data analysis

For data analyses, we used the highest quality position for every duty cycle with a 10 hours on—48 hours off, constant transmission schedule obtained from ARGOS/CLS GPS [13] (S5 Table). We considered juveniles to be independent of their host parents and to have left the breeding area when they were >20 km from the nest [19]. Several birds moved back and forth for some time after independence with no consistent direction of movement. We termed this the pre-migration period and defined it as the period after reaching independence until movement was consistently southward (Table 1, S2 Table). Similar movements are only rarely observed in adults and only one adult in our data set, from the Oulu study site (Fig 1B, S2 Table), performed such pre-migratory movements.

**Table 1:** Timing and duration of autumn migration in common cuckoos.

Parameter	Sample size (adult/juvenile)	Adults	Juveniles
Departure from breeding site	20/5	11 Jul (±11)	26 Jul (±13)
Initiation of migration	20/5	11 Jul (±12)	14 Aug (±24)
Departure NC Europe stopover	18/1	22 Aug (±16)	20 Sep
Leaving Europe	18/1	4 Sep (±14)	29 Sep
Arrival Sahel	16/1	14 Sep (±15)	4 Oct
Arrival winter area	16/1	18 Nov (±22)	3 Dec
Migration duration	16/1	131 (±22)	134
Days from NC Europe stopover to winter ground	16/1	90 (±23)	74
Days from Sahel to winter ground	16/1	64 (±25)	60

Timing (mean date ± SD) and duration (days) of different stages during autumn migration of adult and juvenile cuckoos. NC = North Central. Data on North Swedish adults from [12] and Danish and South Swedish adults from [13].

We defined a stopover as when a bird interrupted migration for more than five days. Individual stopover sites were estimated as average locations within the stationary periods as described in [13]. Due to non-daily transmissions, departure and arrival dates were rarely strictly identifiable, and we used last and first (respectively) days within the region/site. We defined arrival on the winter grounds as when the birds reached the southernmost stationary position.

We calculated initial migration directions using the mean flight directions after individuals crossed a distance of 100 km (Number of juveniles, N_Y_ = 5; Number of adults, N_A_ = 20) from breeding sites. For two juveniles and one adult from Finland, the initial movements were considered pre-migratory movements, but the general direction in which the birds left the breeding grounds was consistent from these movements. The directedness of sample orientations for the individuals of each group was tested with a Rayleigh test. The difference in initial direction between juveniles and adults was tested with a Watson-Williams test (tests based on distances 20–500 km led to similar results except that the directions of juveniles were more scattered at 20 km).

We judged sea-crossing behaviour directly from the maps. Because positions are spaced in time, proof of actual sea-crossings can be hard to obtain. We considered accurate positions over water or islands that connected positions over land on both sides of the Baltic Sea to be proof of a sea-crossing. We estimated potential individual sea-crossing distances as consecutive loxodromic distances between the last location before crossing, and the first location after crossing the Baltic. We subtracted the parts of these distances that occurred over continental land. We also compared the potential sea-crossing distances, irrespective of whether sea-crossing was considered proven or not, between adults and juveniles based on the shortest routes.

Differences in the geometry of juvenile and adult tracks were also tested. First, longitudes of migration routes were compared between the juveniles (N_Y_ = 3) and northern adult cuckoos (N_NA_ = 6) at the southernmost latitude of migration where there were still three juvenile cuckoos migrating (55°N, Fig 1A and 1B). Second, for latitudes ranging from the North European stopovers to the southern edge of Sahel (7°N) where the adults followed a detoured route, we compared route deviations in the juvenile (N_Y_ = 1) and the adults (N_A_ = 10, only from year 2010). We calculated deviations from the direct loxodromic route from breeding to wintering grounds for each individual. Interpolated locations were calculated on the loxodromic routes using the latitudes of the location estimates of each track and the longitudes of the loxodromic routes at each of these latitudes. Route deviations were calculated as the horizontal distance from the location estimates of the tracks to the interpolated locations and tested using a Wilcoxon test. For the migration leg south of the Sahel (7°N), we only compared directions to the winter grounds of juvenile and adult cuckoos (Fig 1D) because there was no detour in adults and other differences could not be tested due to low sample size (N_Y_ = 1; N_A_ = 16).

Evaluation of differences in total migration duration and timing of the different stages for juveniles and adult cuckoos was based on descriptive measures because of low sample sizes (Table 1, S2 Table). We estimated the mean difference and standard deviation in timing based on all pair-wise inter-age-class differences and compared observed mean difference by randomly resampling from pooled adult and juvenile timing (1,000 bootstrap replicates). Potential effects of wind during migration were considered for initial migration as well as for the migration from North-Central Europe to the sub-Sahel (S1 File). We used R 3.1.2 [21] for linear statistics, Oriana 3.0 [22] for circular statistics and diagrams, and the RNCEP package [23] for wind analysis.

## Results

Five juvenile cuckoos were considered to have reached independence and to have departed the breeding grounds (i.e. positions >20 km from the breeding grounds). Of these, two individuals covered considerable distances during pre-migratory movements (1541 and 832 km; Fig 1B). Movement was initially toward southwest and two of three birds leaving Finland evidently crossed the Baltic Sea for extended distances (Fig 1B). The single juvenile cuckoo tag transmitting after the juveniles left Northern Europe revealed straight southward migration from Northern Europe (183°). From Poland this bird stopped over in the Sahel before reaching the wintering area in Angola (Fig 1A).

The initial movement direction of juveniles was 230° (100 km from breeding sites: N = 5, r = 0.98, P = 0.002; Fig 1C), which was significantly different (P < 0.001, F_1, 23_ = 27.8) from the initial direction of all adults of 143° (N = 20, r = 0.86, P < 0.001; Fig 1C). Potential sea-crossing distances in juvenile birds (N = 3, 765 ± 264 km [mean ± SD]; Fig 1B) were longer than those of northern adult birds (N = 6, 263 ± 333 km) that to a large degree circumvented the Baltic Sea, and also longer than those of southern adults (N = 14, 204 ± 46 km). The North-Central European stopover used by the juvenile birds was in the same area as that used by southern adults, which was 600 km west of the area used by northern adults. The stopover of the one juvenile reaching the Sahel was in North-East Nigeria, about 840 km west of that used by adult birds but on the same latitudinal range. The juvenile bird wintered in the same area as the Scandinavian adults, west of where the Finnish adult wintered in Congo (Fig 1A).

In northern Europe, the juveniles (N = 3) migrated further west than the northern adults (N = 6, t = -6.9, df = 7, P < 0.001, two sample t-test). From the North European stopovers to the southern edge of the Sahel, adult birds (N = 10) migrated less directly (for all individuals, positions were significantly, P < 0.001, east of the direct loxodromic route, except for one individual that was not significantly different, P = 0.20) than the juvenile (P = 0.08, N = 10 positions). Positions of the adults were east of those of the juvenile (Fig 1A). After leaving the Sahel, the adult birds changed direction to southwest and the winter grounds (N = 16, α = 200°, r = 0.98, P < 0.001), while the juvenile flew toward 176° (Fig 1D).

**Fig 2:**
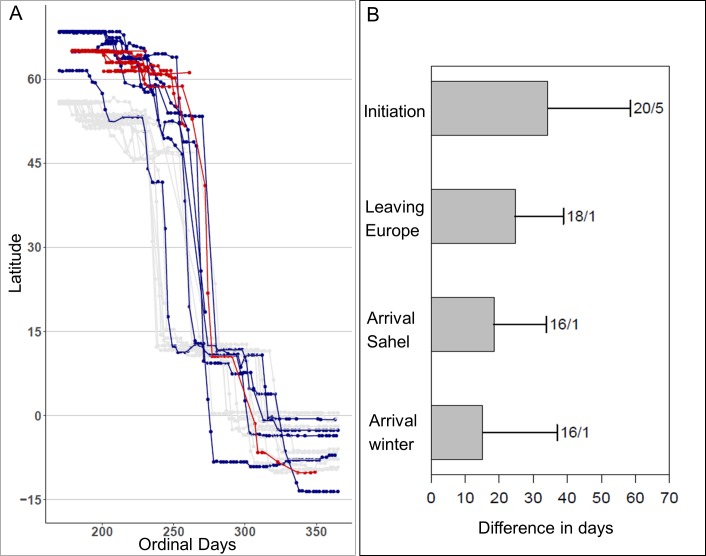
Timing of autumn migration in juvenile and adult common cuckoos. (A) Latitudes traversed by Finnish juveniles (red), northern (blue) and southern adults (grey). Time is from the final part of breeding season until arrival on wintering grounds. (B) Mean difference between juveniles and adults in timing of migration, based on pair-wise inter-age-class differences, at different step latitudes during fall migration (error bars indicate standard deviations; numbers indicate sample size, adults/juveniles). Data on North Swedish adults from [12] and Danish and South Swedish adults from [13].

Migration duration was similar for the juvenile (from initiation to Angola in 134 days) and adults (N = 16 tracks, 131 ± 22 days, 95% CI: 120–141 days). Juveniles timed all migration stages later than adults, on average. For the initiation of migration, there was a clear difference (strongly supported by the bootstrap replicates with fewer than 1 in 1000 showing as large a difference). The juvenile migrated faster after leaving Europe and arrived at a similar time as adults to the final winter grounds (Table 1, S2 Table, Fig 2; no difference in bootstrap replicates). Compared to the adults, initiation of migration was delayed (on average 34 ± 7 days; Table 1) by pre-migratory movements in two juveniles, but the juvenile completing autumn migration showed no pre-migratory movements (S2 Table).

There was no apparent difference in the winds experienced during initial movements (Fig A in S1 File). Both the juvenile and the adults presumably experienced considerable crosswinds during migration (Table A, Figs B and C in S1 File).

## Discussion

This study provides the first detailed tracking data describing the first migration in a smaller, nocturnal long-distance migrant. Our results suggest that young common cuckoos possess an innate migration programme enabling successful migration to wintering grounds independently of adults. The migration pattern of juvenile common cuckoos was similar to that of adults, including stopover and wintering latitudes. In contrast to adults, juveniles left the breeding sites slowly and with less consistently directed movements. Also, their flight directions differed and sea-crossings were more extensive than in adult birds. Migration was more westerly in juvenile birds; initially on a southwest course which later turned south from the stopover in North-Central Europe. Timing of migration for juveniles was later than adults throughout the period, however, after the stopover in North-Central Europe, migration to wintering grounds was completed faster by juveniles, resulting in the arrival at the winter grounds shortly after adults.

Some juvenile cuckoos showed extensive pre-migratory movements that have not been characteristic in adult cuckoos. These pre-migratory movements in the juvenile birds resulted in initial dispersal across vast areas prior to directed migration. Such dispersal may be related to exploration of potential future breeding sites [24], training orientation senses [25–26] or finding future stopover sites [27], but our data do not allow separation of these possibilities.

Juvenile cuckoos also showed extensive sea-crossings, uncharacteristic of migration in adult cuckoos. Extended sea-crossings by juveniles may seem surprising, but assuming that fewer predators are hunting at sea, this could be a predator-avoidance strategy. This driver has been suggested for bar-tailed godwit *Limosa lapponica* [28] with juvenile birds potentially more at risk than adults. However, given the apparent mortality during sea-crossing (two juveniles stopped transmitting during the extended crossings) and the generally shorter crossings in adults, these sea-crossings appear disadvantageous. Similar longer sea-crossings were found in juvenile Eurasian honey buzzards *Pernis apivorus* [29, 30] and Egyptian vultures [20] compared to adults, also with higher apparent mortality for juveniles.

The initial south-westerly orientation fits the migration direction of redstarts, the cuckoo’s local host species. However, juvenile cuckoos apparently migrated before redstarts [31] making it unlikely that the juveniles followed their foster parents. In Europe, migration direction changed clockwise in adults whereas in juveniles it changed counter-clockwise, resulting in southward directed movements in both groups before leaving Europe. It seems unlikely that the different migration in juveniles was caused by them belonging to a separate cuckoo gens, characterised by laying blue eggs, using another migration route [32]. This is supported by the fact that the migration of the two adults, presumably from the same gens as the juveniles, did not differ markedly from the other adults.

The differences in orientation that we found between juveniles and adults imply that young cuckoos change their route in later migrations–likely before their second year since tracks of second year cuckoos show migration route orientation similar to older adults [13]. Age-specific route orientations suggest an adaptive change only available to adults. Age-dependent differences in migration strategies, likely arising from social learning added to the genetic programme, are documented in a few larger, mostly diurnally, migrating species such as the Eurasian honey buzzards *Pernis apivorus* [29, 30].

Our study suggests the potential of juvenile birds to travel straight south even when facing crosswinds. This contrasts with expectations that juveniles are expected to be less able to compensate for crosswinds compared to adults [33]. Juveniles are also generally expected to show more scattered flight directions than adults during autumn migration [13, 27, 29, 34]. Nevertheless, the straight southward directed part of the route and the faster migration of the juvenile cuckoo suggests it relied on inherited directional guiding to reach the wintering area. Potential compensation could be based on simple (sign) navigation using celestial and geomagnetic cues as found in displaced juvenile songbirds [7, 35].

In conclusion, the completed autumn migration of the juvenile cuckoo suggests that the genetic migration programme allowed it to reach the population-specific winter grounds despite traveling in different directions and timing than adults. Life-time tracks of inexperienced birds displaced over a suite of distances are needed to understand whether learning or innate programme or a combination is required for flexibility *en route*.

## Supporting Information

S1 File Supplementary Wind Analysis. (DOCX)

S2 File Tracking Data. (XLSX)

S1 Table Number of ringed common cuckoos per country and country where southernmost recovery was obtained. Ringing periods are shown in the country column. Information obtained from European bird ringing atlases.
(DOCX)

S2 Table Timing and duration of the autumn migration periods of satellite-tracked juvenile and adult cuckoos in 2010–2014. Dates are given for arrivals and departures and the duration of each stage is given in days. ID = Bird identification with sex (F = female, M = male) followed by satellite-tag ID and last two digits of the tagging year in brackets. Age codes follow EURING (3 = First year, 5 = Second year, 6 = After second year, 7 = Third year, 9 = Forth year). Population: DK = Denmark, SS = South Scandinavia, NS = North Scandinavia FI = Finland. N Europe stopover applies only to northern cuckoos and juveniles. NC = North Central. * 62.8°N, ** 58.7°N. The young were sexed based on sex specific genes.
(DOCX)

S3 Table Morphometrics and ages of the young cuckoos. Measured when tagging (N = 13). Tag activation is given as days after tagging.
(DOCX)

S4 Table Fate of tagged cuckoos across studies. Logistic regression (Generalised Linear Model with logit link and binomial error distribution) of Finland post-fledging vs. UK post-fledging: parameter estimate of difference in location: 1.17; SD = 0.70; *z* = 1.68, P = 0.09. Finland juvenile autumn vs. UK adult autumn: parameter estimate of difference in age/location: 1.87; SD = 1.16; *z* = 1.61, P = 0.10.
(DOCX)

S5 Table Location quality of positions included in the study. Number of locations and location quality (LQ A-B, 0–3; CLS 2007–2015) of cuckoos tracked from leaving the breeding areas to arrival on the winter grounds (as the southernmost stationary position) or end of transmission.
(DOCX)

## References

[1] Guilford T, Åkesson S, Gagliardo A, Holland RA, Mouritsen H, Muheim R, et al Migratory navigation in birds: new opportunities in an era of fast-developing tracking technology. J Exp Biol. 2011;214: 3705–12. 10.1242/jeb.051292 22031734

[2] Larkin RP. Spatial distribution of migrating birds and small-scale atmospheric motion In: Papi F, Wallraff HG, editors. Avian navigation. Berlin: Springer; 1982 pp. 28–37.

[3] Zuur B. Nearest neighbour distance in day and night migrating birds. Vogelwarte. 1984;32: 206–18.

[4] Thorup K, Holland RA, Tøttrup AP, Wikelski M. Understanding the Migratory Orientation Program of Birds: Extending Laboratory Studies to Study Free-Flying Migrants in a Natural Setting. Integr Comp Biol. 2010;50: 315–22. 10.1093/icb/icq065 21558206

[5] Bridge ES, Thorup K, Bowlin MS, Chilson PB, Diehl RH, Fléron RW, et al Technology on the move: recent and forthcoming innovations for tracking migratory birds. Bioscience. 2011;61: 689–98.

[6] Mewaldt LR. California sparrows return from displacement to Maryland. Science. 1964;146: 941–2. 10.1126/science.146.3646.941 17777062

[7] Thorup K, Ortvad TE, Rabøl J, Holland RA, Tøttrup AP, Wikelski M. Juvenile songbirds compensate for displacement to oceanic islands during autumn migration. PLoS ONE. 2011;6: e17903 10.1371/journal.pone.0017903 21464975 PMC3064565

[8] Payne R, Christie DA. Common Cuckoo (*Cuculus canorus*) In: del Hoyo J, Elliott A, Sargatal J, Christie DA, de Juana E, editors. Handbook of the Birds of the World Alive. Barcelona: Lynx Edicions; 2016 Available from: http://www.hbw.com/node/54799.

[9] Davies NB. Cuckoos, cowbirds and other cheats. London: T & AD Poyser; 2000.

[10] Sutherland WJ. The heritability of migration. Nature. 1988;334: 471–2.

[11] Seel DC. Migration of the northwestern European population of the cuckoo (*Cuculus canorus*), as shown by ringing. Ibis. 1977;119: 309–22.

[12] Alerstam T, Andersson NÅ, Klaassen R, Olofsson P, Strandberg R, Vardanis J. Gökens okända flytt avslöjad. Vår Fågelvärld. 2015: 38–45.

[13] Willemoes M, Strandberg R, Klaassen RH, Tøttrup AP, Vardanis Y, Howey PW, et al Narrow-front loop migration in a population of the common cuckoo *Cuculus canorus*, as revealed by satellite telemetry. PLoS ONE. 2014;9: e83515 10.1371/journal.pone.0083515 24421890 PMC3885432

[14] Thomson RL, Tolvanen J, Forsman JT. Cuckoo parasitism in a cavity nesting host: near absent egg-rejection in a northern redstart population under heavy apparent (but low effective) brood parasitism. J Avian Biol. 2016;47: 363–70.

[15] Grim T, Rutila J, Cassey P, Hauber ME. The cost of virulence: an experimental study of egg eviction by brood parasitic chicks. Behav Ecol. 2009;20: 1138–46.

[16] Hewson CM, Thorup K, Pearce-Higgins JW, Atkinson PW. Population decline is linked to migration route in the common cuckoo. Nat Commun. 2016; 7. 10.1038/ncomms12296 PMC4960304 27433888

[17] Kleven O, Moksnes A, Røskaft E, Rudolfsen G, Stokke BG, Honza M. Breeding success of common cuckoos *Cuculus canorus* parasitising four sympatric species of *Acrocephalus* warblers. J Avian Biol. 2004;35: 394–8.

[18] Sklepowicz B, Halupka L. The use of sympatric reed warblers *Acrocephalus scirpaceus* and marsh warblers *Acrocephalus palustris* as breeding hosts: parasitism rates and breeding success of common cuckoos *Cuculus canorus*. Acta Ornithol. 2009;44: 177–84.

[19] Wyllie I. The cuckoo. London: Bastford; 1981.

[20] Oppel S, Dobrev V, Arkumarev V, Saravia V, Bounas A, Kret E, et al High juvenile mortality during migration in a declining population of a long‐distance migratory raptor. Ibis. 2015;157: 545–57.

[21] R Development Core Team. R: A language and environment for statistical computing. The R Foundation for Statistical Computing 2011 Available from: http://www.R-project.org/.

[22] Kovach WL. Oriana–Circular Statistics for Windows, ver. 3. Kovach Computing Services. 2009. Available from: http://www.kovcomp.co.uk/oriana/newver3.html.

[23] Kemp MU, van Loon EE, Shamoun-Baranes J, Bouten W. RNCEP: global weather and climate data at your fingertips. Methods Ecol Evol. 2012;3: 65–70.

[24] Morton ML, Wakamatsu MW, Pereyra ME, Morton GA. Postfledging dispersal, habitat imprinting, and philopatry in a montane, migratory sparrow. Ornis Scand. 1991;22: 98–106.

[25] Mukhin A, Kosarev V, Ktitorov P. Nocturnal life of young songbirds well before migration. Proc R Soc Lond B Biol Sci. 2005;272: 1535–39. 10.1098/rspb.2005.3120 PMC1559836 16048767

[26] Brown JM, Taylor PD. Adult and hatch-year blackpoll warblers exhibit radically different regional-scale movements during post-fledging dispersal. Biol Lett. 2015;11: 20150593 10.1098/rsbl.2015.0593 26631243 PMC4707692

[27] Hake M, Kjellén N, Alerstam T. Satellite tracking of Swedish ospreys *Pandion haliaetus*: autumn migration routes and orientation. J Avian Biol. 2001;32: 47–56.

[28] Gill RE, Tibbitts TL, Douglas DC, Handel CM, Mulcahy DM, Gottschalck JC, et al Extreme endurance flights by landbirds crossing the Pacific Ocean: ecological corridor rather than barrier? Proc R Soc Lond B Biol Sci. 2009;276: 447–57. 10.1098/rspb.2008.1142 PMC2664343 18974033

[29] Hake M, Kjellén N, Alerstam T. Age-dependent migration strategy in honey buzzards *Pernis apivorus* tracked by satellite. Oikos. 2003;103: 385–96.

[30] Agostini N. Additional observations of age-dependent migration behaviour in western honey buzzards *Pernis apivorus*. J Avian Biol. 2004;35: 469–70.

[31] Valkama J, Saurola P, Lehikoinen A, Lehikoinen E, Piha M, Sola P, et al Suomen Rengastusatlas 2 [The Finnish Bird Ringing Atlas, Volume 2]. Helsinki: LUOMUS—Finnish Museum of Natural History; 2014.

[32] Fossøy F, Sorenson MD, Liang W, Ekrem T, Moksnes A, Møller AP, et al Ancient origin and maternal inheritance of blue cuckoo eggs. Nat Commun. 2016;7: 10272 10.1038/ncomms10272 26754355 PMC4729921

[33] Thorup K, Alerstam T, Hake M, Kjellén N. Bird orientation: compensation for wind drift in migrating raptors is age dependent. Proc R Soc Lond B Biol Sci. 2003;270: S8–11. 10.1098/rsbl.2003.0014 PMC1698035 12952622

[34] Mellone U, López‐López P, Limiñana R, Piasevoli G, Urios V. The transequatorial loop migration system of Eleonora’s falcon: differences in migration patterns between age classes, regions and seasons. J Avian Biol 2013;44: 417–26.

[35] Åkesson S, Morin J, Muheim R, Ottosson U. Dramatic orientation shift of white-crowned sparrows displaced across longitudes in the high arctic. Curr Biol. 2005;15: 1591–97. 10.1016/j.cub.2005.07.027 16139216

