# Supplementary material for: First-Time Migration in Juvenile Common Cuckoos Documented by Satellite Tracking
Source: PLoS One. 2016 Dec 22;11(12):e0168940. doi: 10.1371/journal.pone.0168940 (PMC5179092; doi:10.1371/journal.pone.0168940)
Supplement: S1 File — (DOCX) [file pone.0168940.s001.docx]

**S1 File. Supplementary Wind Analysis**

Potential effects of winds on initial travel directions (crossing 100 km distance) in juveniles (N = 5) and adults (N = 20), respectively, were tested using cross- and tail-wind components at surface level. The winds experienced by juveniles and adults were similar for the initial travel (unequal variances t-test for cross-wind: t = 0.2, df = 8.2, P = 0.82; for tail-wind t = -0.2, df = 8.8, P = 0.83, Fig A).

To investigate the ability to cope with crosswinds during the autumn journey, we evaluated spatiotemporal wind patterns during the periods of travel. Wind components were compared in both the juvenile and the adult cuckoos to assess whether the juvenile bird corrected its route in response to wind conditions from North-Central Europe to the sub-Sahel. Comparisons were made for three different parts of the journey, within Europe, Mediterranean – North Sahel and Sahel – Central Africa rainforest, corresponding to different latitude intervals (Tables A and B).

During the period when the cuckoos crossed from North-Central Europe to the winter area, easterly winds (at pressure level 925 mb) prevailed with north-south component (Table A, Fig B). The differences between the crosswind directions along each crossing (Table A) suggest that the juvenile compensated more than the adults (Fig 1). The juvenile bird experienced stronger easterly winds than adults but slightly more tail-winds. More westerly winds only across Europe and the directions of migration show that the adults drifted more than the juvenile during this stage. Similarly across the Sahel and the rainforest, the adults seemed to drift and the juvenile compensated. Maximum east-west and north-south components were higher when crossing Europe and Mediterranean – North Sahel and minimum wind speed was lower at the end of the juvenile migration (Table B). The winds on the day of maximum and minimum wind components are represented in the Fig C for the juvenile migration.

|  |  |  | Adults | | |  | Juvenile | | |  | Juvenile  (narrow longitude range) | | |
| --- | --- | --- | --- | --- | --- | --- | --- | --- | --- | --- | --- | --- | --- |
| Crossing | Latitudes (°N) |  | Uwind | abs uwind | vwind |  | uwind | abs uwind | vwind |  | uwind | abs uwind | vwind |
| Europe | 50–35 |  | 0.7 | 3.2 | -1.8 |  | 2.2 | 3.6 | -2 |  | 2.4 | 3.5 | -1.9 |
| Mediterranean –  North Sahel | 35–17.5 |  | -0.6 | 3 | -3.3 |  | -1.7 | 2.9 | -5.1 |  | -4.5 | 4.9 | -2.5 |
| Sahel – CA rainforest | 17.5–2.5 |  | -0.1 | 2.1 | 0.5 |  | -1.6 | 2.3 | -1.2 |  | -1.6 | 2.7 | -1 |

**Table A. Average wind during autumn migration of the juvenile and adult cuckoos grouped by latitude.** Values are mean east-west wind component (uwind), mean east-west wind strength (absolute uwind: abs uwind) and mean north-south wind component (vwind) in m s–1. The year used for adults was 2010 as it was the most representative (N = 7 until the Sahel and N = 6 for sub-Saharan crossing). The longitudinal range was 12.5°–36.5°E. The narrow longitudinal range (12.5°–17.5°E) of the juvenile migration is given to compare both results for the juvenile, only differing more along the mid-crossing. Equal absolute values of mean east-west wind component and mean east-west wind strength indicate consistency in the crosswind direction. CA = Central Africa.

|  |  |  | uwind | |  | vwind | |
| --- | --- | --- | --- | --- | --- | --- | --- |
| Crossing | Latitudes (°N) |  | Minimum | Maximum |  | Minimum | Maximum |
| Europe | 50–35 |  | 2 | 5.6 |  | 1.2 | 6.2 |
| Mediterranean –  North Sahel | 35–17.5 |  | 3.7 | 6.1 |  | 1.8 | 3.5 |
| Sahel – CA rainforest | 17.5–2.5 |  | 1.2 | 4.7 |  | 0.6 | 3.3 |

**Table B. Minimum and maximum wind during the juvenile autumn migration grouped by latitude.** Values are absolute east-west (uwind) and north-south (vwind) wind components in m s–1. The longitudinal range was 12.5°–17.5°E. CA = Central Africa.

**
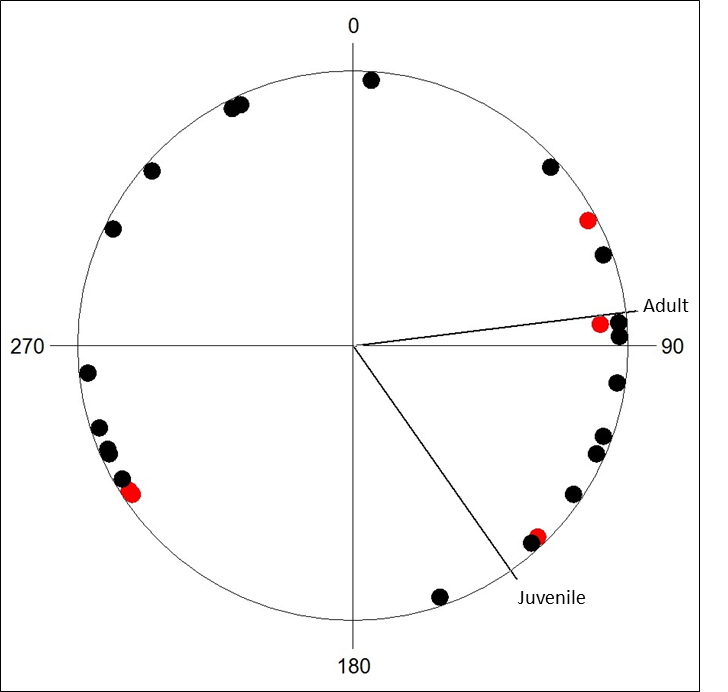
**

**Fig A. Wind direction at initiation of migration.** Wind direction of juvenile (red) and adult (black) cuckoos when crossing 100 km distance (taken as mean of the locations before and after crossing 100 km distance). Mean directions of juvenile (N = 5, α = 145°, concentration r = 0.31, P = 0.64) and adults (N = 20, α = 83°, r = 0.06, P = 0.93) are shown.


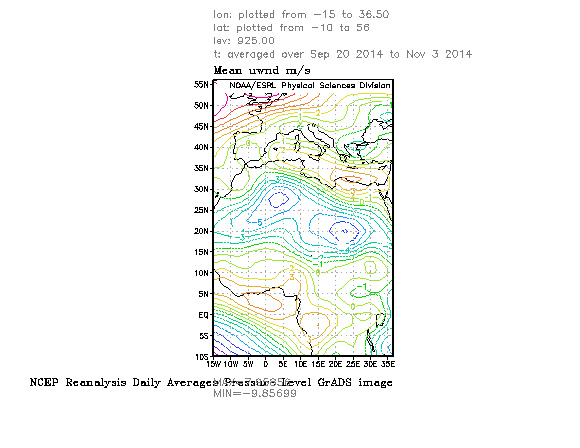

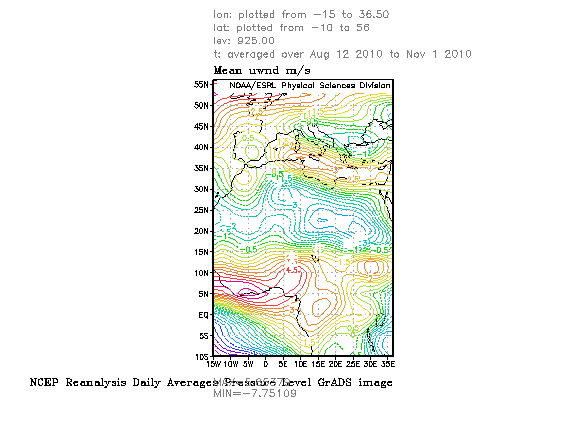


**
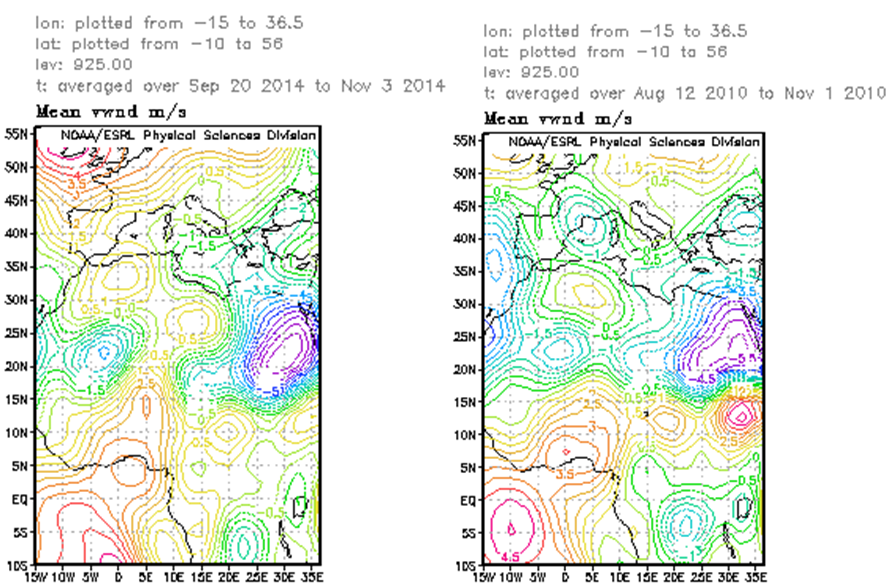
**

**Fig B. Wind direction and strength during autumn migration.** Mean crosswind (uwind; east-west wind component; upper row) and tailwind (vwind; north-south component; lower row) in m s–1 during the period when the juvenile (left panel) and adults (right; data for 2010), respectively, crossed from NC Europe to Sahel. Figures generated at, and data retrieved from, the PSD Web site are in the public domain and may be downloaded freely. They appreciate acknowledgement of the Physical Sciences Division, ESRL (NOAA) in the use of the images and data. This may be done by including text such as: Image provided by Physical Sciences Division, Earth System Research Laboratory, NOAA, Boulder, Colorado, from their Web site at <http://www.esrl.noaa.gov/psd/>.

**(a)**

**
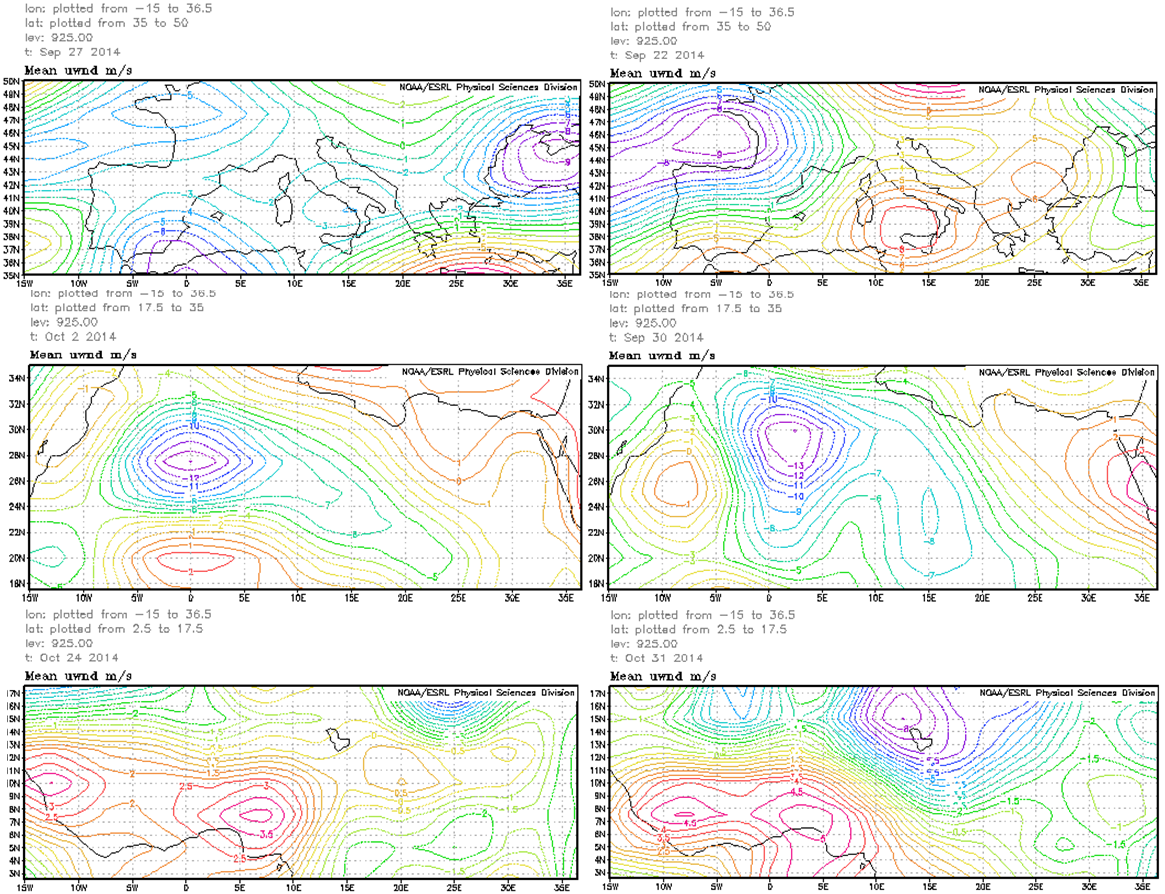
**

**(b)**

**
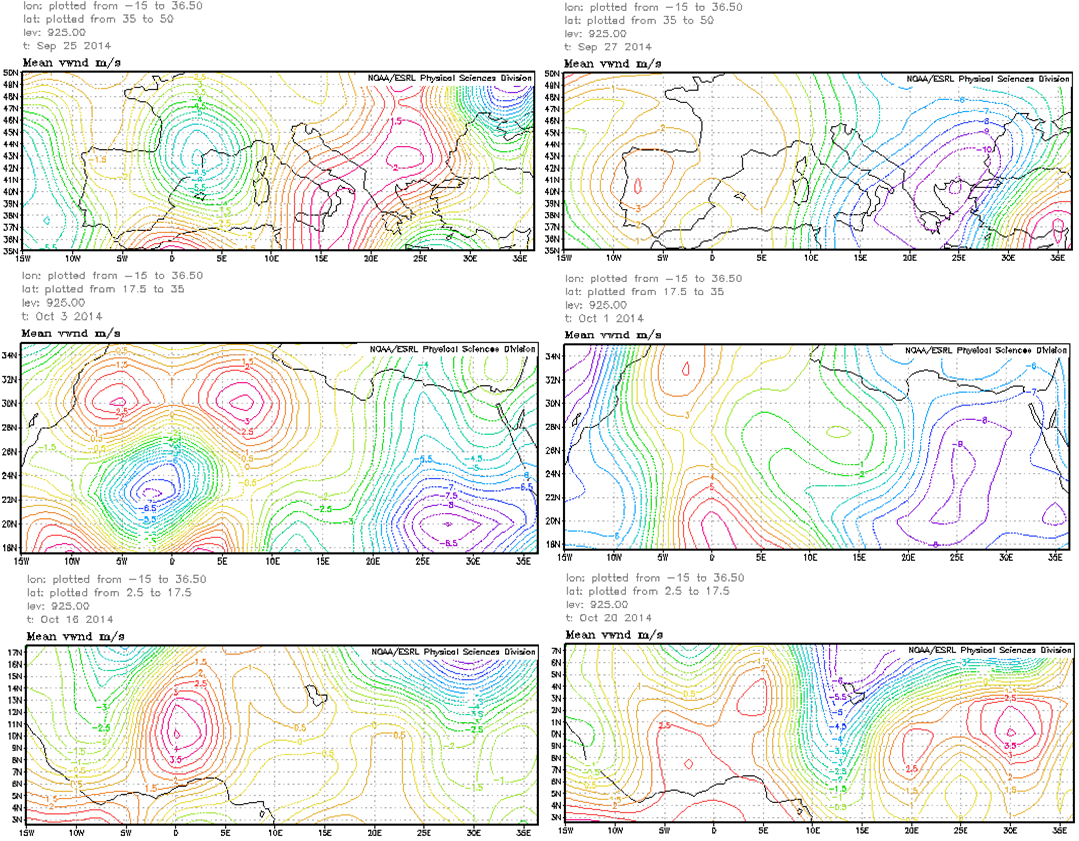
**

**Fig C. Minimum and maximum winds during autumn migration.** Days of average minimum (left) and maximum (right) wind in m s–1 during NC Europe–Sahel crossing of the juvenile (longitudinal range 12.5°–17.5°). (a) crosswinds (uwind) and (b) tailwinds (vwind). Figures generated at, and data retrieved from, the PSD Web site are in the public domain and may be downloaded freely. They appreciate acknowledgement of the Physical Sciences Division, ESRL (NOAA) in the use of the images and data. This may be done by including text such as: Image provided by Physical Sciences Division, Earth System Research Laboratory, NOAA, Boulder, Colorado, from their Web site at <http://www.esrl.noaa.gov/psd/>.
