# Supplementary material for: First-Time Migration in Juvenile Common Cuckoos Documented by Satellite Tracking
Source: PLoS One. 2016 Dec 22;11(12):e0168940. doi: 10.1371/journal.pone.0168940 (PMC5179092; doi:10.1371/journal.pone.0168940)
Supplement: S1 Table — Ringing periods are shown in the country column. Information obtained from European bird ringing atlases. (DOCX) [file pone.0168940.s003.docx]

| Country | Numbers ringed | Southernmost recovery | Sub-Saharan recoveries |
| --- | --- | --- | --- |
| Norway (1914 – 2000) | 416 | Italy | 0 |
| Finland (1974 – 2012) | 2462 | Malta | 0 |
| Sweden (1911 – 2000) | 1312 | Tunisia | 0 |
| Denmark (1898 – 2002) | 566 | Italy | 0 |
| UK (1909 – 1997) | 5894 | Cameroon | 1 |
| Germany (1945 – 2012) | 5019 | Malta | 0 |
| The Netherlands (1911 – 1982) | 44 | Togo | 1 |
| Czech Republic and Slovakia (1934 – 2002) | 1986 | Slovakia | 0 |
| Hungary (1951 –2006) | 615 | Greece | 0 |
| Italy (1928 – 2003) | 935 | Italy | 0 |

**S1 Table. Number of ringed common cuckoos per country and country where southernmost recovery was obtained.** Ringing periods are shown in the country column. Information obtained from European bird ringing atlases.

References:

Bakken V, Runde O, Tjørve E. Norsk Ringmerkingsatlas 2[Norwegian Bird Ringing Atlas, Volume 2]. Stavanger: MUST – Stavanger Museum; 2003.

Valkama J, Saurola P, Lehikoinen A, Lehikoinen E, Piha M, Sola P et al. Suomen Rengastusatlas 2 [The Finnish Bird Ringing Atlas, Volume 2]. Helsinki: LUOMUS – Finnish Museum of Natural History; 2014.

Fransson T, Österblom H, Hall-Karlsson S. Svensk Ringmärkningsatlas 2 [Swedish Bird Ringing Atlas, Volume 2]. Stockholm; 2008.

Bønløkke J, Madsen, JJ, Thorup K, Pedersen KT, Bjerrum M, Rahbek C. Dansk Trækfugleatlas [The Danish Bird Migration Atlas]. Humlebæk: Rhodos; 2006.

Wernham CV, Toms MP, Marchant JH, Clark JA, Siriwardena GM, Baillie SR. The Migration Atlas: movements of the birds of Britain and Ireland. London: Poyser; 2002.

Bairlein F, Dierschke J, Dierschke V, Salewski V, Geiter O, Hüppop K, Köppen U, Fiedler W. Atlas des Vogelzugs - Ringfunde deutscher Brut- und Gastvögel [German Bird Migration Atlas]. Wiebelsheim: AULA-Verlag GmbH; 2014.

Speek BJ, Speek G. Thieme's Vogeltrekatlas [Dutch Bird Migration Atlas]. Zutphen: Thieme & Cie; 1984.

Cepák J, Klvana P, Formanek J, Horak Z, Jelinek M, Schropfer M. Atlas migrace ptáku Ceské a Slovenské Republiky [Czech and Slovak Bird Migration Atlas]. Praha: Aventinum; 2008.

Csorgo T, Karcza Z, Halmos G. Magyar madarvonulasi atlasz [Hungarian Bird Migration Atlas]. Budapest: Kossuth Kiadó; 2009.

Spina F, Volponi S. Atlante della Migrazione degli Uccelli in Italia [Italian Bird Migration Atlas, Volume 1]. Roma: ISPRA – MATTM; 2008.
