# Supplementary material for: First-Time Migration in Juvenile Common Cuckoos Documented by Satellite Tracking
Source: PLoS One. 2016 Dec 22;11(12):e0168940. doi: 10.1371/journal.pone.0168940 (PMC5179092; doi:10.1371/journal.pone.0168940)
Supplement: S2 Table — Dates are given for arrivals and departures and the duration of each stage is given in days. ID = Bird identification with sex (F = female, M = male) followed by satellite-tag ID and last two digits of the tagging year in brackets. Age codes follow EURING (3 = First year, 5 = Second year, 6 = After second year, 7 = Third year, 9 = Forth year). Population: DK = Denmark, SS = South Scandinavia, NS = North Scandinavia FI = Finland. N Europe stopover applies only to northern cuckoos and juveniles. NC = North Central. * 62.8°N, ** 58.7°N. The young were sexed based on sex specific genes. (DOCX) [file pone.0168940.s004.docx]

|  | Bird Information | | | | Timing | | | | | | | | Duration | | | |
| --- | --- | --- | --- | --- | --- | --- | --- | --- | --- | --- | --- | --- | --- | --- | --- | --- |
|  | ID | Age | Breeding Location | Population | Dep breeding site | Initiation of migration | Arr N Europe 67.5°N | Dep N Europe 63.3°N | Dep NC Europe 49.8°N | Dep Europe 36°N | Arr Sahel 18°N | Arr winter area | Premigratory movement | N Europe –  winter ground | NC Europe –  winter ground | Total migration |
| Southern adults | F 36328 (10) | 5 | North Sealand | DK | 17-Jul | 17-Jul | … | … | 27-Jul | 6-Sep | 25-Sep | 23-Oct | 0 | … | 88 | 98 |
|  | F 36328 (11) | 7 | North Sealand | DK | 11-Jul | 11-Jul | … | … | 8-Aug | 11-Sep | 17-Sep | 18-Oct | 0 | … | 71 | 99 |
|  | F 36328 (12) | 9 | North Sealand | DK | 1-Jul | 1-Jul | … | … | … | … | … | … | 0 | … | … | … |
|  | M 36331 (10) | 5 | North Sealand | DK | 4-Jul | 4-Jul | … | … | 11-Aug | 30-Aug | 2-Sep | 11-Dec | 0 | … | 122 | 159 |
|  | M 36331 (11) | 7 | North Sealand | DK | 9-Jul | 9-Jul | … | … | 19-Aug | 19-Aug | 27-Aug | 17-Nov | 0 | … | 90 | 131 |
|  | M 36331 (12) | 9 | North Sealand | DK | 24-Jun | 24-Jun | … | … | 20-Aug | 25-Aug | 28-Aug | 12-Nov | 0 | … | 84 | 140 |
|  | F 36332 (10) | 5 | North Sealand | DK | 29-Jun | 29-Jun | … | … | … | … | … | … | 0 | … | … | … |
|  | M 36487 (10) | 6 | North Sealand | DK | 29-Jun | 29-Jun | … | … | 17-Aug | 17-Aug | 31-Aug | 19-Nov | 0 | … | 94 | 143 |
|  | M 36487 (11) | 6 | North Sealand | DK | 8-Jul | 8-Jul | … | … | 18-Aug | 21-Aug | 26-Aug | 25-Nov | 0 | … | 99 | 140 |
|  | M 36487 (12) | 6 | North Sealand | DK | 27-Jun | 27-Jun | … | … | 9-Aug | 23-Aug | 31-Aug | 21-Nov | 0 | … | 104 | 146 |
|  | M 19150 (10) | 6 | Scania | SS | 11-Jul | 11-Jul | … | … | 17-Aug | 17-Aug | … | … | 0 | … | … | … |
|  | M 49466 (10) | 6 | Scania | SS | 7-Aug | 7-Aug | … | … | 18-Aug | 14-Sep | 20-Sep | 24-Dec | 0 | … | 129 | 140 |
|  | F 57372 (10) | 5 | Scania | SS | 3-Jul | 3-Jul | … | … | 2-Aug | 24-Sep | 9-Oct | 30-Nov | 0 | … | 120 | 150 |
|  | M 57374 (10) | 5 | Scania | SS | 30-Jun | 30-Jun | … | … | 27-Aug | 27-Aug | 29-Sep | 6-Nov | 0 | … | 71 | 128 |
| Northern adults | M 129611 (14) | 5 | Ruokolahti | FI | 8-Jul | 8-Jul | … | … | 18-Aug | 30-Aug | 3-Sep | 5-Oct | 0 | … | 48 | 90 |
|  | M 135532 (14) | 6 | Oulu | FI | 14-Jul | 28-Jul | … | … | 17-Sep | 19-Sep | … | … | 11 | … | … | … |
|  | M Ma65 (11) | 5 | Lapland | NS | 21-Jul | 21-Jul | 23-Jul | 19-Aug | 27-Aug | 15-Sep | 20-Sep | 9-Dec | 0 | 111 | 104 | 141 |
|  | M Ma70 (11) | 6 | Lapland | NS | 15-Jul | 15-Jul | 22-Jul | 11-Aug | 11-Sep | 23-Sep | 28-Sep | 9-Nov | 0 | 90 | 58 | 117 |
|  | M Ma73 (11) | 5 | Lapland | NS | 16-Jul | 16-Jul | 24-Jul | 20-Aug | 27-Sep | 27-Sep | 7-Oct | 19-Dec | 0 | 121 | 83 | 156 |
|  | M Ma73 (12) | 7 | Lapland | NS | 30-Jul | 30-Jul | 4-Aug | 8-Sep | 10-Sep | 10-Sep | 30-Sep | 18-Nov | 0 | 71 | 69 | 111 |
| Young | F 135529 (14) | 3 | Oulu | FI | 23-Jul | 23-Jul | 28-Jul* | 13-Sep** | 20-Sep | 29-Sep | 4-Oct | 3-Dec | 0 | … | 74 | 134 |
|  | M 135528 (14) | 3 | Oulu | FI | 26-Jul | 8-Sep | … | … | … | … | … | … | 44 | … | … | … |
|  | M 135526 (14) | 3 | Oulu | FI | 15-Jul | 5-Sep | … | … | … | … | … | … | 51 | … | … | … |
|  | F 135527 (14) | 3 | Oulu | FI | 18-Jul | 18-Jul | … | … | … | … | … | … | 0 | … | … | … |
|  | M 129612 (13) | 3 | Ruokolahti | FI | 17-Aug | 17-Aug | … | … | … | … | … | … | 0 | … | … | … |

**S2 Table. Timing and duration of the autumn migration periods of satellite-tracked juvenile and adult cuckoos in 2010–2014.** Dates are given for arrivals and departures and the duration of each stage is given in days. ID = Bird identification with sex (F = female, M = male) followed by satellite-tag ID and last two digits of the tagging year in brackets. Age codes follow EURING (3 = First year, 5 = Second year, 6 = After second year, 7 = Third year, 9 = Forth year). Population: DK = Denmark, SS = South Scandinavia, NS = North Scandinavia FI = Finland. N Europe stopover applies only to northern cuckoos and juveniles. NC = North Central. * 62.8°N, ** 58.7°N. The young were sexed based on sex specific genes (Griffiths R, Double MC, Orr K, Dawson RJG. A DNA test to sex most birds. Mol Ecol. 1998;7: 1071–1075. Bantock TM, Prys-Jones RP, Lee PML. New and improved molecular sexing methods for museum bird specimens. Mol Ecol Resour. 2008;8: 519–528).
