# Supplementary material for: First-Time Migration in Juvenile Common Cuckoos Documented by Satellite Tracking
Source: PLoS One. 2016 Dec 22;11(12):e0168940. doi: 10.1371/journal.pone.0168940 (PMC5179092; doi:10.1371/journal.pone.0168940)
Supplement: S3 Table — Measured when tagging (N = 13). Tag activation is given as days after tagging. (DOCX) [file pone.0168940.s005.docx]

| Parameter | Mean ± SD |
| --- | --- |
| Age (days) | 18 ± 2 |
| Wing chord (mm) | 122 ± 9 |
| Body mass (g) | 102 ± 10 |
| Tag activation (days) | 0.9 ± 0.8 |

**S3 Table. Morphometrics and ages of the young cuckoos**. Measured when tagging (N = 13). Tag activation is given as days after tagging.
