# Supplementary material for: First-Time Migration in Juvenile Common Cuckoos Documented by Satellite Tracking
Source: PLoS One. 2016 Dec 22;11(12):e0168940. doi: 10.1371/journal.pone.0168940 (PMC5179092; doi:10.1371/journal.pone.0168940)
Supplement: S4 Table — Logistic regression (Generalised Linear Model with logit link and binomial error distribution) of Finland post-fledging vs. UK post-fledging: parameter estimate of difference in location: 1.17; SD = 0.70; z = 1.68, P = 0.09. Finland juvenile autumn vs. UK adult autumn: parameter estimate of difference in age/location: 1.87; SD = 1.16; z = 1.61, P = 0.10. (DOCX) [file pone.0168940.s006.docx]

| Location | Period | Tagged | Lost | Survival | Reference |
| --- | --- | --- | --- | --- | --- |
| Finland | Post-fledging | 12 | 7 | 0.42 | This study |
| UK | Post-fledging | 26 | 10 | 0.62 | Wyllie 1981 |
| Finland | Juvenile autumn | 5 | 4 | 0.20 | This study |
| S Scandinavia | Adult autumn | 8 | 2 | 0.75 | Willemoes *et al.* 2014 |
| N Scandinavia | Adult autumn | 3 | 1 | 0.67 | Alerstam *et al.* 2015 |
| Finland | Adult autumn | 2 | 1 | 0.50 | This study |
| UK Eastern | Adult autumn | 23 | 8 | 0.65 | Hewson *et al.* 2016 |
| UK Western | Adult autumn | 19 | 9 | 0.53 | Hewson *et al.* 2016 |
| Spain | Post-fledging great spotted cuckoo | 38 | 14 | 0.63 | Soler *et al.* 1994* |

**Table S4. Fate of tagged cuckoos across studies**. Logistic regression (Generalised Linear Model with logit link and binomial error distribution) of Finland post-fledging vs. UK post-fledging: parameter estimate of difference in location: 1.17; SD = 0.70; *z* = 1.68, P = 0.09. Finland juvenile autumn vs. UK adult autumn: parameter estimate of difference in age/location: 1.87; SD = 1.16; *z* = 1.61, P = 0.10.

*Soler M, Palomino JJ, Martinez JG. Activity, survival, independence and migration of fledgling great spotted cuckoos. Condor. 1994;96: 802–805.
