# Supplementary material for: First-Time Migration in Juvenile Common Cuckoos Documented by Satellite Tracking
Source: PLoS One. 2016 Dec 22;11(12):e0168940. doi: 10.1371/journal.pone.0168940 (PMC5179092; doi:10.1371/journal.pone.0168940)
Supplement: S5 Table — Number of locations and location quality (LQ A-B, 0–3; CLS 2007–2015) of cuckoos tracked from leaving the breeding areas to arrival on the winter grounds (as the southernmost stationary position) or end of transmission. (DOCX) [file pone.0168940.s007.docx]

| LQ | Number of locations |
| --- | --- |
| 3 | 296 |
| 2 | 211 |
| 1 | 133 |
| 0 | 40 |
| A | 109 |
| B | 102 |

**S5 Table. Location quality of positions included in the study.** Number of locations and location quality (LQ A-B, 0-3; CLS 2007–2015) of cuckoos tracked from leaving the breeding areas to arrival on the winter grounds (as the southernmost stationary position) or end of transmission.
